# Proprioceptive Based Training for stroke recovery. Proposal of new treatment modality for rehabilitation of upper limb in neurological diseases

**DOI:** 10.1186/s40945-015-0007-8

**Published:** 2015-08-03

**Authors:** Pawel Kiper, Alfonc Baba, Michela Agostini, Andrea Turolla

**Affiliations:** 1 Laboratory of Kinematics and Robotics, IRCCS San Camillo Hospital Foundation, via Alberoni 70, Venice, 30126 Italy; 2 Department of Neuroscience, University of Sheffield, Glossop Road, Sheffield, S10 2JF UK

**Keywords:** Stroke, Neurorehabilitation, Upper limb, Bilateral arm training, Intracortical inhibition

## Abstract

**Background:**

The central nervous system (CNS) has plastic properties allowing its adaptation through development. These properties are still maintained in the adult age and potentially activated in case of brain lesion. In the present study authors hypothesized that a significant recovery of voluntary muscle contraction in post stroke patients experiencing severe upper limb paresis can be obtained, when proprioceptive based stimulations are provided. Proprioceptive based training (PBT) is based on performing concurrent movements with both unaffected and affected arm, with the aim to foster motor recovery through some mutual connections of interhemispheric and transcallosal pathways. The aim of this pre-post pilot study was to evaluate the feasibility of PBT on recovery of voluntary muscle contraction in subacute phase after stroke.

**Methods:**

The treatment lasted 1 h daily, 5 days per week for 3 weeks. The PBT consisted of multidirectional exercises executed synchronously with unaffected limb and verbal feedback. The Medical Research Council scale (MRC), Dynamometer, Fugl-Meyer Upper Extremity scale (F-M UE), Functional Independence Measure scale (FIM) and modified Ashworth scale were administered at the beginning and at the end of training. Statistical significance was set at *p* < 0.05.

**Results:**

Six patients with severe paresis of the upper limb within 6 months after stroke were enrolled in the study (5 ischemic and 1 hemorrhagic stroke, 3 men and 3 women, mean age 65.7 ± 8.7 years, mean distance from stroke 4.1 ± 1.5 months) and all of them well tolerated the training. The clinical changes of voluntary muscle contraction after PBT were statistically significant at the MRC scale overall (*p* = 0.028), and dynamometer assessment overall (*p* = 0.028). Each patient improved muscle contraction of one or more muscles and in 4 out of 6 patients voluntary active movement emerged after therapy. The functional outcomes (i.e. F-M UE and FIM) did not show significant change within group.

**Conclusions:**

The findings of this preliminary research revealed that PBT may be a feasible intervention to improve the motricity of upper limb in stroke survivors.

## Background

The impairment of motor function represents one of the major causes of disability after stroke. More than 69 % of cerebrovascular lesions provokes impairment of motor function to the upper limb, moreover about 56 % of subjects experience severe hemiparesis even after 5 years from stroke [1, 2]. The functional impairment of the upper limb can impact negatively on quality of life and limit the autonomy in many activities of daily living (ADL). The recovery process occur through neural mechanisms mediating spontaneous cortical reorganization, but evidence indicates that intensive stimulation is essential to improve motor recovery either [3–7]. Several studies, both in animals and humans, have shown different reorganization of the central nervous system (CNS), at molecular as well as synaptic connectivity levels, dependent on the type of behavioral interactions with the external environment [4, 7]. The possibility to estimate as early as possible the outcomes achievable due to rehabilitation is one of the most meaningful clinical point to address, with the aim to promote the best recovery after stroke. This information is important for prognosis and personalization of rehabilitation programs for every individual patient [8, 9]. Several modalities for upper limb motor rehabilitation after stroke were proposed recently, among that the bilateral training has received outstanding attention. The bilateral training is a method based on the execution of repetitive tasks with both (affected and non–affected) upper extremities, with the aim of regaining a better motor function. This approach is based on the rationale that bilateral movements allow to optimize the activation/inhibition balance between the two hemispheres. The bilateral motor training emphasizes the importance of bilateral approach, intended as the involvement of both limbs in daily life practice (e.g. dressing, bathing, feeding, driving, cooking). Several studies on healthy subjects showed that when a motor task is performed the dominant arm has the goal to reach the target, whereas the non-dominant arm play a role in stabilizing adjacent musculoskeletal districts [10]. Evidence from studies with transcranial magnetic stimulation (TMS) in stroke survivors showed that both hemispheres induce a reduction of intra-cortical inhibition, when bilateral movements were performed. On the contrary, if only one arm was activated the increased inhibition was observed in the ipsilateral hemisphere [10, 11]. However, it is not clear whether the modifications in cortical excitability are attributable to the non-use of the affected limb or to compensatory overuse of the healthy limb [12]. These findings raise the hypothesis that the reduction of excitability of the healthy hemisphere can help to improve motor function of the paretic limb after stroke.

The conventional neuromotor rehabilitation for upper limbs early after stroke in case of complete plegia, consists mainly of passive mobilization or electrical stimulation, since most of the current available therapies require that residual voluntary activation of muscles or partial movements are present [13, 14]. Most of the studies on bilateral arm training focused their attention on the functional improvement of the affected limb and in most of them the motor training is provided by robotic devices, performing passive movement of affected arm and active movement of non-affected limb [15, 16]. The current findings did not report a meaningful advantage on functional outcomes for the affected limb treated with bilateral training [17]. However, clinical studies with functional magnetic resonance imaging (fMRI) seemed to confirm the importance of bilateral exercises to improve motor learning [5]. Therefore, to understand exhaustively which recovery mechanisms occur after stroke it is fundamental that new therapeutic modalities are tested in clinical settings. The proprioceptive based training (PBT) aims to stimulate the emergence of voluntary contraction and is based on motor learning principles, such as the repetition of tasks with concurrent use of feedbacks. The proposed concept is based on the concurrent repetitions of movements performed with the non-affected limb and with the affected one, passively mobilized by physiotherapist in charge of guaranteeing the optimal kinematic execution. Executing the intention to move in the affected limb, supported by physiotherapist, aims to re-educate the proprioceptive sensitivity coherently to each movement phase. Proprioception is the capacity of CNS to determine where all of body parts are positioned at any given time. Proprioceptors located in soft tissues can sense changes and pass afferent information to the brain. Hypothetically PBT can reinforce proprioception through simple movement executed in one plane where any compensatory movements (e.g. shoulder rotation) are abolished. Furthermore, the treatment modality requires recognition of position of both limbs which can act on affected arm proprioception. The common application of bilateral arm training (BAT), is based only on passive movements supported by robotic devices and the potential active movement of affected limb is not permitted. Therefore the difference between PBT and BAT is that the movement in the unaffected limb is performed synchronously with patient’s efferent motor command to both limbs, as assisted by physiotherapist on affected side. Thus, the treatment requires generation of voluntary activation from patient, as reference for both limbs, moreover physiotherapist is asked to adapt passive mobilization of affected limb simultaneously with voluntary active movements of unaffected one. The recognition of position of both limbs, therefore, is always requested. The advantage of the proposed approach relies on the possibility to be applied since the early acute phase after stroke, when several therapeutic modalities (e.g. Constraint-Induced Movement Therapy) cannot be provided because of the absence of residual voluntary muscular activation. Moreover, according to Whitall et al. the involvement of unaffected limb in bilateral training represents a fundamental component for training, based on the rationale of the interlimb coupling theory, where stimuli from two limbs concur to create a “neurofunctional” unit [18].

The purpose of this pre-post pilot study was to evaluate the feasibility of PBT for fast recovery of voluntary muscle contraction, after stroke. The end-point of the proposed treatment modality was based on mean force increase of at least 20 % of all the muscles considered for the PBT treatment.

## Methods

### Recruitment

All the patients were informed about the aim and procedures of the study and written informed consent was obtained from all the participants. The pre-post pilot study group included inpatients affected by a first stroke (i.e. ischemic or hemorrhagic) occurred no longer than 6 months before the enrolment (mean distance from onset 4.1 ± 1.5 months). Presence of severe upper limb paresis (0 to 1 point according to the Medical Research Council scale) of the following muscles: deltoid, biceps brachii, triceps brachii, flexor carpi radialis, flexor carpi ulnaris, extensor carpi radialis, extensor carpi ulnaris, flexor digitorum and extensor digitorum; passive range of motion (ROM) completely free; absence of primary joint trauma of the wrist, elbow and shoulder, were considered as inclusion criteria. All patients who refused to participate were excluded from the study together with ones presenting: increased muscle tone defined as a score higher than 1 point (modified Ashworth scale) in at least one of the treated muscles described above for MRC assessment, apraxia (De Renzi test < 62 points) [19], global sensory aphasia (clinical notes), neglect (clinical notes), cognitive impairments (Mini Mental State Examination MMSE < 24 points) [20], sensitivity disorders (defined as < 2 points in items shoulder, elbow, wrist and thumb at the proprioceptive sensitivity section of the Fugl-Meyer scale), stroke lesion located in the cerebellum (clinical notes). The institutional review board of the IRCCS San Camillo Hospital Foundation (Italy) approved the study protocol (Prot. 2012.07 BAT v.1.2) and the study have been conducted in accordance with the Declaration of Helsinki.

### Interventions

During the treatment patient was lying supine with the upper limbs positioned in symmetric posture. The subject was asked to move both limbs with the same frequency performing bilateral flexion-extension of one of the upper limb districts according to the available free ROM of the target joint. The movement execution of the affected arm was supported by the physiotherapist performing the passive movement at the same rhythm, as the one executed with the unaffected side. During the therapeutic session patient was asked to focus his/her attention on the movement performed against gravity, which was reinforced by a verbal command. Afterwards, the physiotherapist fully supported movement execution coherently with the patient’s movement initialization. The active movements performed voluntarily by the patient with unaffected limb were considered as the reference movement, that the physiotherapist has to emulate passively, by synchronization of passive movement executed in phase with the affected side. The treatment lasted one hour and was divided as follows: 2 proprioceptive based stimulation sessions per 3 min for each movement, with a rest of 2 min between every session. Every patient received 15 treatments, 5 days a week, for 3 weeks. The PBT was introduced as additional hour to conventional neuromotor treatment (CNT). Each patient received CNT, based on tailored individual exercises (passive, active-assisted or active), in accordance with the patient’s functional status and with the aim to: reduce degree of disability, improve quality of life. The patients performed exercises for postural control in sitting and standing position, exercises for coordination with and without physiotherapist assistance and gait training, whether appropriate.

However, the upper limb motion were trained providing only PBT and passive mobilization, to maintain mechanical properties of soft tissues.

### Clinical outcome measures

The assessment was conducted by two physiotherapists not involved in providing the experimental treatment. The following domains were assessed at the beginning and at the end of treatment: muscles strength, motor impairment and functional activities. The primary outcomes were: the Medical Research Council (MRC) scale [21] and force measured by dynamometer. The following muscles were considered: deltoid, biceps brachii, triceps brachii, flexor carpi radialis, flexor carpi ulnaris, extensor carpi radialis, extensor carpi ulnaris, flexor digitorum and extensor digitorum. The secondary outcomes were: the Fugl-Meyer upper extremity (F-M UE) scale [22] for upper limb motor function, the Functional Independence Measure (FIM) scale for global independence [23], the modified Ashworth scale for spasticity [24]. Positions for dynamometer test were standardized as requested for MRC scale assessment. Data were recorded on study case report sheet form, which contained the patient’s study number, date of enrolment, pre and post assessment results, adverse events, and clinical data. Completed form was registered on study management database.

### Statistical analysis

The descriptive results were reported as mean and standard deviation. Moreover, distribution skewness was assessed by Shapiro-Wilk test and due to the small sample size non-parametric test for paired measures (Wilcoxon) was used to determine the differences before and after treatment. Statistical significance level was set at *p* < 0.05 and IBM SPSS 20.0 package software was used for the analysis.

## Results

A group of 28 eligible patients was screened between July 2013 and October 2014. Among them 21 did not meet the inclusion criteria and 1 did not complete the full training session. According to the inclusion/exclusion criteria 6 patients were enrolled for PBT (3 men and 3 women), to evaluate the feasibility of treatment. The group consisted of 5 ischemic and 1 hemorrhagic stroke, the mean age was 65.7 ± 8.7 years, and the mean distance from stroke was 4.1 ± 1.5 months. All patients reported to be comfortable throughout the training. Table 1 represents motor and functional mean score of the affected arm of the 6 patients before and at the end of PBT treatment. The voluntary muscle contraction of the biceps brachii, flexor carpi radialis and flexor digitorum (MRC scale) significantly improved after PBT. Furthermore, as measured by dynamometer the muscle strength of biceps brachii significantly increased after PBT. The results of the study showed that each patient improved muscle contraction in one or more muscles and in 4 out of 6 patients the full range of voluntary active movement emerged after therapy, mainly in elbow flexion and extension (Table 2). Moreover, in 4 out of 6 patients the active movement relieved by physiotherapist appeared for following movements: shoulder flexion, elbow flexion, wrist flexion and extension. The F-M UE scale, the FIM scale and the modified Ashworth scale did not change significantly after treatment.

**Table 1:** Effect of proprioceptive based training on overall outcomes

Clinical outcomes	Pre-test	Post-test	*p*-value
MRC	0.16 ± 0.15	0.98 ± 0.36	.028*
Dynamometer	1.36 ± 1.81	6.72 ± 3.48	.028*
F-M UE	4.50 ± 1.22	5.16 ± 1.32	.285
FIM	68.33 ± 32.26	75.16 ± 27.05	.066
MAS	3.00 ± 4.28	3.16 ± 4.26	.564

Data are displayed as mean and standard deviation

*MRC* Medical Research Council, *F*-*M UE* Fugl-Meyer Upper Extremity, *FIM* Functional Independence Measure, *MAS* Modified Ashworth Scale

*p < 0.05; Wilcoxon test

**Table 2:** Effect of proprioceptive based training on single muscles

Clinical outcomes	Patient 1		Patient 2		Patient 3		Patient 4		Patient 5		Patient 6		Overall		*p*
	Before	After	Before	After	Before	After	Before	After	Before	After	Before	After	Before	After	
MRC															
*Deltoid*	0	2	1	2	0	0	1	1	0	2	0	1	0.33 ± 0.51	1.33 ± 0.81	.063
*Biceps brachii*	0	3	1	2	0	1	1	2	0	3	1	3	0.50 ± 0.54	2.33 ± 0.81	.026*
*Triceps brachii*	0	3	1	3	0	0	0	0	0	3	0	1	0.16 ± 0.40	1.16 ± 1.50	.066
*Flexor carpi radialis*	0	2	0	1	0	1	1	1	0	1	0	1	0.16 ± 0.40	1.16 ± 0.40	.034*
*Flexor carpi ulnaris*	0	2	0	1	0	0	0	0	0	0	0	1	0.00 ± 0.00	0.66 ± 0.81	.102
*Extensor carpii radialis*	0	0	0	0	0	0	0	1	0	0	0	1	0.00 ± 0.00	0.33 ± 0.51	.157
*Extensor carpi ulnaris*	0	0	0	0	0	0	0	0	0	0	0	1	0.00 ± 0.00	0.16 ± 0.40	.317
*Flexor digitorum*	0	1	0	1	1	1	0	1	0	0	0	1	0.16 ± 0.40	0.83 ± 0.40	.046*
*Extensor digitorum*	0	0	0	0	1	1	0	0	0	0	0	1	0.16 ± 0.40	0.33 ± 0.51	.317
Dynamometer															
*Deltoid*	0	15	7	17	0	0	0	0	0	12	0	8	1.16 ± 2.85	8.60 ± 7.37	.068
*Biceps brachii*	0	31	7	19	0	7	13	21	0	27	8	16	6.26 ± 7.48	20.21 ± 8.49	.028*
*Triceps brachii*	0	26	12	31	0	0	0	0	0	39	0	7	3.33 ± 8.16	17.26 ± 17.0	.068
*Flexor carpi radialis*	0	14	0	0	0	6	0	0	0	12	0	9	0.00 ± 0.00	6.88 ± 5.97	.068
*Flexor carpi ulnaris*	0	14	0	0	0	0	0	0	0	0	0	6	0.00 ± 0.00	3.26 ± 5.71	.180
*Extensor carpi radialis*	0	0	0	0	0	0	0	0	0	0	0	4	0.00 ± 0.00	0.66 ± 1.63	.317
*Extensor carpi ulnaris*	0	0	0	0	0	0	0	0	0	0	0	4	0.00 ± 0.00	0.66 ± 1.63	.317
*Flexor digitorum*	0	5	0	0	5	5	0	0	0	0	0	4	0.83 ± 2.04	2.26 ± 2.53	.180
*Extensor digitorum*	0	0	0	0	4	4	0	0	0	0	0	0	0.66 ± 1.63	0.66 ± 1.63	1.000

Data are reported as mean and standard deviation (m ± SD)

*MRC* Medical Research Council, *p < 0.05; Wilcoxon test

## Discussion

To our knowledge, this study is the first one assessing the effect of specific proprioceptive stimulation provided for one hour of training, in stroke patients. Both, results achieved and patients’ positive feedback after experimental treatment suggest the feasibility of proposed intervention modality. Moreover, patients in this study showed improvement of muscle force after intervention. The significant changes observed mainly in the biggest muscles (e.g. elbow flexion/extension, actuated by biceps and triceps brachii, respectively) could be due to the easiness of stimulating large muscles acting in one main axis than small muscles involved in many complex movements. Our findings showed that PBT may induce a recovery of voluntary muscle contraction in the subacute phase after stroke. Moreover, several muscles (i.e. deltoid, triceps brachii, flexor carpi radialis and ulnaris) showed clinical improvement. The lack of reaching statistical significance could be due to the small sample of patients, as well as to the many outcomes collected. The functional scales (i.e. F-M UE and FIM) did not show differences within group, however, the functional outcomes of common used bilateral training were tested before and previous evidence showed mixed results for them [17]. This finding sustains our hypothesis that PBT could play a role in the first phase after stroke, by promoting a better background for following rehabilitation therapies. These preliminary results are important for sustaining the possibility to treat patients beneficially soon after stroke, in fact muscle contraction is the basic prerequisite for expressing an effective motor behavior. Our preliminary findings indicate that motor training, whether enriched by proprioceptive exercises involving unaffected arm, should be introduced in the subacute phase of rehabilitation after stroke, as a clinical tool to elicit physiological muscular contraction. Furthermore, the absence of changes at the modified Ashworth scale suggests that the training did not increase pathological muscle tone. As mentioned previously, the largest repertoire of known rehabilitation therapies require the presence of minimum voluntary motor activation which is a limitation for their application in the acute or subacute phase after stroke. The possibility to approach patients experiencing an almost complete paralysis of the limb, with PBT may provide a motor background useful for future functional and complex rehabilitation modalities. Results of this study allow to hypothesize that simultaneous stimulation of both afferent and efferent pathways enriched by verbal feedback may play a role in the creation of new connections in the affected brain structures [5]. Indeed, during the treatment patient create efferent command action, conversely afferent information is provided through the execution of assisted movement, in the affected limb. On this basis, concomitant movements may act on both signals concurrently and might act directly on some of the mechanisms exploiting the transcallosal pathways.

Several studies suggest that inter-hemispheric connection between M1s plays a main role for the control of hand movements [25, 26]. Studies on stroke patients reported that the excitability of M1 in unaffected hemisphere is augmented after stroke, because of a lack of inhibition from the stroke affected hemisphere [25, 27, 28]. The bilateral movement studied with fMRI showed increased activation in both side of the cerebellum and some plastic changes in both hemispheres. Conversely, decreased activation has been noted in cerebellum when unilateral movement was provided. Moreover, the fMRI studies showed that the cerebellum could be a critical site involved in bilateral movement [5].

In multicenter study from Platz et al., authors compared unilateral approach for impairment-oriented training, with conventional therapy. Authors showed that the patients can benefit from highly structured therapeutic intervention and they concluded that specificity of training can be more important for arm motor recovery, than intensity [29].

The results of our study showed that the PBT could be a favorable approach for motor recovery, after stroke. Future studies are needed with the aim to optimize the technical aspects of the intervention, moreover the implications in a larger cohort of patients enrolling various clinical pictures has to be explored.

### Limitations of the study and further research perspectives

Some limitations as to be acknowledged, the changes observed within outcomes perhaps were due to the combination of CNT and PBT, because both were introduced in this study. The time since stroke could be an issue inasmuch the spontaneous recovery is the main driving factor in subacute phase, after stroke. The voluntary muscle contraction observed after training was statistically significant, however, the results were not clinically meaningful for functional outcomes as resulted with absence of statistical significance. Therefore, both small sample size and absence of a control group are further limitations of this study. The authors’ future work will be focused to increment the sample and to compare results with a control group, with the aim to test the potential effectiveness of PBT.

## Conclusions

With respect to previous proposals of bilateral arm training, this study extended its application to subacute phase after stroke, including a highly structured program enriched by proprioceptive exercises. Furthermore, the modality proposed aims to underline the importance of muscles contraction for upper limb recovery. Therefore, it is important to provide to clinicians an useful tool, which could be easily introduced into rehabilitation programme. Despite the small group, results has shown an effect of the PBT in improving muscle contraction and some aspects of motor control on the biggest muscles of the upper limb. These preliminary results on PBT feasibility need to be addressed in future larger controlled studies, to consider its implementation in real clinical settings.
